# Effects of exogenous GnRH administration at insemination on pregnancy rates of estrus-synchronized seven ewe populations during the breeding season

**DOI:** 10.1590/1984-3143-AR2024-0085

**Published:** 2025-02-24

**Authors:** Shuyuan Sun, Nana Yang, Jing Zhang, Xinglong Wu, Yiyong Liu, Xiangyun Li

**Affiliations:** 1 College of Animal Science and Technology, Hebei Technology Innovation Center of Cattle and Sheep Embryo, Hebei Agricultural University, Baoding, Hebei, China; 2 Institute of Xinjiang Yili Animal Science, Yining, Xinjiang, China

**Keywords:** GnRH, timed artificial insemination, pregnancy rate, litter size, sheep

## Abstract

The objective of this study was to investigate the effects of GnRH at insemination on pregnancy and lambing in seven ewe populations during the breeding season. Estrus was synchronized in 1560 adult ewes using an intravaginal sponge impregnated with flurogestone acetate. The sponge was left in the vagina for 12 days followed by an injection of 330 IU of eCG at sponge removal. Each ewe was inseminated twice at 48 h and 60 h after sponge removal. The treatment group was intramuscularly injected at the first insemination with a dose of 16 μg GnRH and the control group with saline solution in each ewe population. The results showed that GnRH administration significantly decreased the pregnancy rate in three ewe populations, but had no effects in four ewe populations. Additionally, the litter size tended to increase in the treatment group compared to the control group in all seven ewe populations, but the difference was not significant. In conclusion, GnRH administration at insemination was not recommended for ewes undergoing a timed artificial insemination during the breeding season. The breed/population may be a critical determinant of the potential for exploiting GnRH application in sheep breeding programs.

## Introduction

The combination of artificial insemination and synchronization of estrus, commonly called timed artificial insemination (TAI) without estrus detection, is an important and practical technique in the sheep industry. It can increase lamb production and reproductive efficiencies, improve flock management and add genetic improvement (Türk et al., 2008; Gibbons et al., 2019). The most commonly used protocols for estrus synchronization in ewes are based on treatment with intravaginal devices of progesterone release or intravaginal sponges impregnated with fluorogestone acetate or medroxyprogesterone acetate, associated with equine chorionic gonadotropin (eCG) near the end of progestogen treatment (Menchaca and Rubianes, 2004; Titi et al., 2010). In traditional treatments, progestogens are used for 12-14 days periods similar to the lifespan of a cyclic corpus luteum, regardless of the stage of the cycle or the follicular status of the ovary at the time of treatment initiation (Gibbons et al., 2019; Hameed et al., 2021).

An extensive body of literature has shown that gonadotropin hormone-releasing hormone (GnRH) can be useful to synchronize and ensure ovulation in farm animal reproduction (Menchaca and Rubianes, 2004; Gonźalez-Bulnes et al., 2004; Reyna et al., 2007; Azawi and Al-Mola, 2011; Balaro et al., 2016). Although GnRH is already widely used in ovine TAI programs (Hameed et al., 2021), its effects on pregnancy rates are controversial (Menchaca and Rubianes, 2004). Some studies reported that a single dose of GnRH given 24 to 48 h after pessary removal increased the pregnancy rate (Biehl et al., 2017; Kutlu and Dinç, 2021), while some others have reported no changes (Reyna et al., 2007; Türk et al., 2008; Olivera-Muzante et al., 2011; Cavalcanti et al., 2012; Ayaseh et al., 2021) or even decreased the pregnancy rate (Walker et al., 1989; Martemucci and D'Alessandro, 2011; Olivera-Muzante et al., 2013). Based on these previous studies, it is suggested that the effects of GnRH on pregnancy rates are strongly dependent on the ewe breed/population. The objective of this study was to investigate the effects of exogenous GnRH administration at the time of artificial insemination on pregnancy rates in seven ewe populations synchronized with progestagen-eCG combination during the breeding season.

## Methods

### Animals

This study was conducted in Yining of Ili Kazak Autonomous Prefecture of the Xinjiang Uygur Autonomous Region, Chengde of Hebei, and Xilingol League of the Inner Mongolia Autonomous region in China during the breeding season (September - December). A total of 1560 pluriparous and non-lactating ewes (3 - 6 years old with 40 - 60 kg body weight and 150-180 days postpartum) and forty-six adult rams (3 - 5 years old with 70 - 90 kg body weight, consisting of ten Dorper rams, twelve Australian White rams, sixteen Texel rams, and eight Suffolk rams) were used. The ewes consisted of seven populations: Huyang (n = 201), Dorper × Huyang cross-bred (n = 160), Hanyang (n = 187), Australian White × Hanyang cross-bred (n = 240), Kazak (n = 246), Texel × Kazak cross-bred (n = 300), Mongolian (n = 226), and were inseminated with Dorper, Dorper, Australian White, Australian White, Texel, Texel, and Suffolk rams, respectively. During the experimental period, the ewes were kept away from the rams to prevent voluntary mating. All ewes were kept indoors and fed a diet of corn, wheat straw, and alfalfa hay, supplemented with grass. Fresh drinking water and salt bars were provided *ad libitum*. No nutritional flushing was applied to the animals before mating. The authors declare that all procedures in the experiment were approved by the Research Ethics Committee of Hebei Agricultural University (#202045) and were conducted in ways consistent with animal welfare guidelines.

### Semen preparation

Semen was collected with an artificial vagina (Muqimuye Sci-Tech Co., Ltd., Shanghai, China) from rams and all the ejaculates were within acceptable parameters regarding volume (0.7-2.0 mL), spermatozoa concentration (> 3 × 10^9^ spermatozoa per mL), and spermatozoa motility (> 70% progressive motility). The ejaculates were pooled and diluted in ultra-high temperature-treated commercial skimmed milk (YILI^TM^, YILI Group, China) to reach a concentration of 400 × 10^6^ spermatozoa cells per mL. Spermatozoa motility was assessed using hemocytometer slides under a light microscope. The semen was kept at 30°C in a water bath until insemination.

### Estrus synchronization and insemination

Each ewe was treated with a polyurethane intravaginal sponge impregnated with 45 mg of flurogestone acetate (Muqimuye Sci-Tech Co., Ltd., Shanghai, China) for 12 days followed by an injection of 330 IU of eCG i.m. (Sansheng Biological Technology Co., Ltd., Ningbo, China) at sponge removal. Each ewe was subjected to cervical insemination twice at 48 (± 2) h and 60 (± 2) h after sponge removal using an insemination device (Zhengmu Bio-Tech Co., Ltd, Baoding, China) containing 0.25 ml of diluted semen (approximately 100 × 10^6^ spermatozoa). The ovine cervical opening was located using a speculum and a head light. Each ewe was restrained by raising the hind limbs against a thick stick. The perineal area was scrubbed with antiseptic soap and warm water thoroughly. A speculum was inserted into the vagina and pushed against the tissue surrounding the cervix to help center the external cervical os with the aid of a head light. The device was inserted into the cervix, and the semen was slowly released as deep as possible into the cervix. Semen dilution and insemination were performed by two experienced technicians. Each ewe population was divided into the treatment group and the control group (Table 1). The treatment group was subjected to intramuscular administration of 16 μg of the GnRH agonist triptorelin (Sansheng Biological Technology Co., Ltd., Ningbo, China) at the first insemination, while the control group received 1 mL of sterile physiological saline solution.

**Table 1:** Effects of GnRH on pregnancy and lambing in seven estrus-synchronized ewe populations Ewes in the treatment group and the control group were subjected at the time of the first insemination to intramuscular administration of 16 μg of the GnRH agonist triptorelin and 1 mL of sterile physiological saline solution, respectively. Pregnancy rate = Number of lambing ewes/Number of inseminated ewes × 100; Litter size = Number of total lambs/Number of lambing ewes; a, b: the difference between values with different letters in the same column is significant at the *p* value.

**Populations**	**Groups**	**Number of sponge in**	**Number of sponge out**	**Number of inseminated ewes**	**Pregnancy rate (%)**	**Litter size**
Huyang	Control	101	101	101	81.2%(82/101)	2.28
	Treatment	100	98	98	79.6%(78/98)	2.36
Dorper × Huyang	Control	80	78	78	76.9%(60/78)	1.80
	Treatment	80	80	80	76.3%(61/80)	1.95
Hanyang	Control	93	92	90	75.6%(68/90)	2.04
	Treatment	94	93	93	72.0%(67/93)	2.21
Australia White × Hanyang	Control	120	119	119	67.2%(80/119)	2.03
	Treatment	120	117	117	64.1%(75/117)	2.17
Kazak	Control	122	120	120	71.7%(86/120)^a^	1.05
	Treatment	124	122	121	56.2%(68/121)^b^	1.10
Texel × Kazak	Control	150	147	147	69.4%(102/147)^a^	1.16
	Treatment	150	148	148	56.1%(83/148)^b^	1.27
Mongolian	Control	90	90	90	77.8%(70/90)^a^	1.28
	Treatment	136	136	135	62.2%(84/135)^b^	1.35

### Data analysis

The pregnancy rate (number of lambing ewes /number of inseminated ewes) and litter size (number of lambs/number of lambing ewes) were calculated after all deliveries were completed. The data were statistically analyzed using SAS Version 8.0. The pregnancy rate and litter size were analyzed using the Chi-squared test. It was considered significant if the calculated *p* values were less than 0.05.

## Results

The values concerning pregnancy rates and litter size in the control and treatment groups of seven ewe populations were shown in Table 1. The pregnancy rates in the control group and the treatment group in Huyang, Dorper × Huyang cross-bred, Hanyang, and Australian White × Hanyang cross-bred populations were 81.2% *vs* 79.6%, 76.9% *vs* 76.3%, 75.6% *vs* 72.0%, and 67.2% *vs* 64.1%, respectively, and were not significantly different (*p* > 0.05). However, the pregnancy rates in the control group were significantly higher compared to the treatment group in Kazak (71.7% *vs* 56.2%, *p* = 0.0124), Texel × Kazak cross-bred (69.4% *vs* 56.1%, *p* = 0.0181), and Mongolian (77.8% *vs* 62.2%, *p* = 0.0139) populations. The litter size in the control group and the treatment group in Huyang, Dorper × Huyang cross-bred, Hanyang, Australian White × Hanyang cross-bred, Kazak, Texel × Kazak cross-bred, and Mongolian populations were 2.28 *vs* 2.36, 1.80 *vs* 1.95, 2.04 *vs* 2.21, 2.03 *vs* 2.17, 1.05 *vs* 1.10, 1.16 *vs* 1.27, 1.28 *vs* 1.35, respectively. The litter size tended to increase in the treatment group compared to the control group in all seven ewe populations, but there were no significant differences (*p* > 0.05).

## Discussion

The aim of this study was to evaluate the effects of GnRH given at the time of artificial insemination on the pregnancy rate in estrus-synchronized seven ewe populations undergoing a TAI procedure during the breeding season. According to our data, GnRH administration significantly decreased the pregnancy rate in Kazak, Texel × Kazak cross-bred, and Mongolian populations, but had no effects in Huyang, Dorper × Huyang cross-bred, Hanyang, and Australian White × Hanyang cross-bred populations. This suggested that the effects of GnRH on pregnancy rates are strongly dependent on the breed/population of the ewe. One possible explanation for the decreased pregnancy rate in Kazak, Texel × Kazak cross-bred, and Mongolian populations after GnRH treatment was that the induced abnormal uterine environment impairs embryonic implantation and development. In the present study, due to sponge loss, the number of ewes at sponge insertion was different from the number of ewes at sponge removal in some populations. Two, one, and one ewes were eliminated at insemination due to vaginal infection. In previous studies performed in ewes during the non-breeding season, a single dose of GnRH administration between day of estrus and 49 h after removal of the progestagen pessary was reported to increase the pregnancy rate by 2.0% - 30.1% (Kutlu and Dinç, 2021), while Ishida et al. (1999) reported an 11.7% decrease in the pregnancy rate compared to its own control group. In estrus-synchronized Awassi ewes after short-term progestagen administration, a dose of 50 μg of GnRH (Gonodarelin diacetate) given 48 h after removal of the progestagen pessary decreased the pregnancy rate to 37.9% compared to 55.2% in its own control group (Kutlu and Dinç, 2021). Some other studies, in which injection of a single dose of GnRH (24 h-48 h after removal of the progestagen pessary/on the day of estrus) was performed during the breeding season, reported varying degrees of positive responses from 4% to 73.2% in pregnancy rate (Walker et al., 1989; Sirjani et al., 2012; Kutlu and Dinç, 2021), while some others have reported no changes (Türk et al., 2008; Olivera-Muzante et al., 2011; Cavalcanti et al., 2012; Ayaseh et al., 2021) or decrease in pregnancy rate (Olivera-Muzante et al., 2013). Moreover, Biehl et al. (2017) reported that the use of GnRH was efficient in increasing the pregnancy rate in Santa Ines ewes undergoing the timed artificial insemination procedure. The best protocol for GnRH administration was at the time of insemination, as only three managements were required. Additionally, a dose of 25 μg of GnRH agonist triptorelin given 48 h after sponge removal significantly decreased the pregnancy rates of Suffolk ewes and Ujimqin ewes subjected to the timed artificial insemination procedure during both the breeding and non-breeding season but did not significantly decrease the pregnancy rate of Small-tailed Han ewes during the non-breeding seasons (unpublished data). Further research is needed to investigate the effects of GnRH administration at insemination on pregnancy and lambing in seven different ewe populations during the non-breeding season. For labor management, the pregnancy rate was calculated at lambing and equal to the number of ewes lambing divided by the number of ewes inseminated, instead of using ultrasonic diagnosis in the present study. These data suggested that the breed/population may be a critical determinant of the potential for the exploitation of GnRH application in ovine TAI programs.

GnRH can be useful to synchronize and ensure ovulation (Reyna et al., 2007; Azawi and Al-Mola, 2011; Balaro et al., 2016). A single dose of GnRH given 24 - 36 h after removal of the progestagen pessary synchronizes the LH peak approximately 40 h after pessary withdrawal and ovulation occurs in 90% of ewes within 72 h after pessary withdrawal (Menchaca and Rubianes, 2004). Moreover, a single dose of GnRH given at the end of a superstimulation protocol induces an LH peak within 4 h and ovulation occurs between 20 and 32 h later (Walker et al., 1986, 1989; Rubianes and Menchaca, 2003; Reyna et al., 2007; Martemucci and D'Alessandro, 2011). Menchaca et al. (2009) reported that the administration of GnRH 24 h after removal of the progestagen pessary significantly improved the fertilization rate, ovulation rate, and number of transferable embryos per treated donor ewe. These previous studies indicated that a single dose of GnRH given at insemination may increase litter size (Cam et al., 2002; Türk et al., 2008; Fernandez et al., 2019; Kutlu and Dinç, 2021). This was in accordance with the present study, in which GnRH administration tended to increase litter size in the seven ewe populations.

## Conclusion

Although GnRH administration tended to increase litter size, the pregnancy rate did not increase in four ewe populations and decreased in three ewe populations in this study. These data strongly suggested that GnRH administration at insemination is not recommended for ewes undergoing the timed artificial insemination procedure during the breeding season. The breed/population may be a critical determinant of the potential for exploiting GnRH application in sheep breeding programs.

## References

[B001] Ayaseh M, Mirzaei A, Boostani A, Mehrvarz M (2021). The effect of prostaglandin and gonadotrophins (GnRH and hCG) injection combined with the ram effect on progesterone concentrations and reproductive performance of Karakul ewes during the non-breeding season. Vet Med Sci.

[B002] Azawi OI, Al-Mola MK (2011). A study on the effect of GnRH administration on the ovarian response and laparoscopic intrauterine insemination of Awassi ewes treated with eCG to induce superovulation. Trop Anim Health Prod.

[B003] Balaro MF, Fonseca JF, Barbosa TG, Souza-Fabjan JM, Figueira LM, Teixeira TA, Carvalheira LR, Brandão FZ (2016). Potential role for GnRH in the synchronization of follicular emergence before the superovulatory Day 0 protocol. Domest Anim Endocrinol.

[B004] Biehl MV, Ferraz MVC, Ferreira EM, Polizel DM, Miszura AA, Barroso JPR, Oliveira GB, Bertoloni AV, Pires AV (2017). Effect of reproductive methods and GnRH administration on long-term protocol in Santa Ines ewes. Trop Anim Health Prod.

[B005] Cam MA, Kuran M, Yildiz S, Selcuk E (2002). Fetal growth and reproductive performance in ewes administered GnRH agonist on day 12 post-mating. Anim Reprod Sci.

[B006] Cavalcanti A, Brandao F, Nogueira L, Fonseca J (2012). Effects of GnRH administration on ovulation and fertility in ewes subjected to estrous synchronization. Rev Bras Zootec.

[B007] Fernandez J, Bruno-Galarraga MM, Soto AT, de la Sota RL, Cueto MI, Lacau-Mengido IM, Gibbons AE (2019). Effect of GnRH or hCG administration on Day 4 post insemination on reproductive performance in Merino sheep of North Patagonia. Theriogenology.

[B008] Gibbons A, Bruno-Galarraga M, Fernandez J, Gonzalez-Bulnes A, Cueto M (2019). Vitrified embryo transfer in Merino sheep under extensive conditions. Anim Reprod.

[B009] Gonźalez-Bulnes A, Baird DT, Campbell BK, Cocero MJ, García-García RM, Inskeep EK, López-Sebastián A, McNeilly AS, Santiago-Moreno J, Souza CJ, Veiga-López A (2004). Multiple factors affecting the efficiency of multiple ovulation and embryo transfer in sheep and goats. Reprod Fertil Dev.

[B010] Hameed N, Khan MI, Zubair M, Andrabi SMH (2021). Approaches of estrous synchronization in sheep: developments during the last two decades: a review. Trop Anim Health Prod.

[B011] Ishida N, Okada M, Sebata K, Minato M, Fukui Y (1999). Effects of GnRH and hCG treatments for enhancing corpus luteum function to increase lambing rate of ewes artificially inseminated during the non-breeding season. J Reprod Dev.

[B012] Kutlu M, Dinç DA (2021). The effect of double-dose GnRH injections on reproductive performance parameters following short-term progestagen administration in lactated Awassi ewes during the non-breeding season. Trop Anim Health Prod.

[B013] Martemucci G, D’Alessandro AG (2011). Synchronization of oestrus and ovulation by short time combined FGA, PGF(2α), GnRH, eCG treatments for natural service or AI fixed-time. Anim Reprod Sci.

[B014] Menchaca A, Rubianes E (2004). New treatments associated with timed artificial insemination in small ruminants. Reprod Fertil Dev.

[B015] Menchaca A, Vilariño M, Pinczak A, Kmaid S, Saldaña JM (2009). Progesterone treatment, FSH plus eCG, GnRH administration, and Day 0 Protocol for MOET programs in sheep. Theriogenology.

[B016] Olivera-Muzante J, Fierro S, López V, Gil J (2011). Comparison of prostaglandin- and progesterone-based protocols for timed artificial insemination in sheep. Theriogenology.

[B017] Olivera-Muzante J, Gil J, Viñoles C, Fierro S (2013). Reproductive outcome with GnRH inclusion at 24 or 36h following a prostaglandin F2α-based protocol for timed AI in ewes. Anim Reprod Sci.

[B018] Reyna J, Thomson PC, Evans G, Maxwell WM (2007). Synchrony of ovulation and follicular dynamics in merino ewes treated with GnRH in the breeding and non-breeding seasons. Reprod Domest Anim.

[B019] Rubianes E, Menchaca A (2003). The pattern and manipulation of ovarian follicular growth in goats. Anim Reprod Sci.

[B020] Sirjani MA, Kohram H, Shahir MH (2012). Effects of eCG injection combined with FSH and GnRH treatment on the lambing rate in synchronized Afshari ewes. Small Rumin Res.

[B021] Titi HH, Kridli RT, Alnimer MA (2010). Estrus synchronization in sheep and goats using combinations of GnRH, progestagen and prostaglandin F2α. Reprod Domest Anim.

[B022] Türk G, Gür S, Sönmez M, Bozkurt T, Aksu EH, Aksoy H (2008). Effect of exogenous GnRH at the time of artificial insemination on reproductive performance of Awassi ewes synchronized with progestagen–PMSG–PGF2α combination. Reprod Domest Anim.

[B023] Walker SK, Smith DH, Ancell P, Seamark RF (1989). Time of ovulation in the South Australian Merino ewe following synchronization of estrus. 2. Efficacy of GnRH treatment and its relevance to insemination programs utilizing frozen-thawed semen. Theriogenology.

[B024] Walker SK, Smith DH, Seamark RF (1986). Timing of multiple ovulations in the ewe after treatment with FSH or PMSG with and without GnRH. J Reprod Fertil.

